# Completeness Evaluation of Adult-Population-Based Cancer Registries: A Systematic Review

**DOI:** 10.3390/cancers17071123

**Published:** 2025-03-27

**Authors:** Mariana P. Sousa, Teresa Monjardino, Cristina Costa Santos, Lúcio Lara, Maria José Bento

**Affiliations:** 1 Epidemiology, Outcomes, Economics and Management in Oncology Group—Portuguese Oncology Institute of Porto Research Center (CI-IPOP), Porto Comprehensive Cancer Center (Porto.CCC) & RISE@CI-IPOP (Health Research Network), 4200-072 Porto, Portugal; mariana.p.sousa@ipoporto.min-saude.pt (M.P.S.); monjardino.teresa@gmail.com (T.M.); 2 Experimental Pathology and Therapeutics Group—Research Center, Porto Comprehensive Cancer Center (Porto.CCC) & RISE@CI-IPOP (Health Research Network), Portuguese Oncology Institute of Porto (IPO Porto), 4200-072 Porto, Portugal; lucio.santos@ipoporto.min-saude.pt; 3 Department of Community Medicine, Information and Health Decision Sciences (MEDCIDS), Faculty of Medicine, University of Porto, 4200-319 Porto, Portugal; csantos@med.up.pt; 4 Departamento de Ciências Médicas, Universidade de Aveiro, 3810-193 Aveiro, Portugal; 5 CINTESIS@RISE, Faculty of Medicine, University of Porto, 4200-319 Porto, Portugal; 6 Surgical Oncology Department, Portuguese Institute of Oncology of Porto, 4200-072 Porto, Portugal; 7 Department of Population Studies, ICBAS-School of Medicine and Biomedical Sciences, University of Porto, 4050-313 Porto, Portugal

**Keywords:** accuracy, completeness, data quality, population-based cancer registry, quality standards

## Abstract

Population-based cancer registries are crucial for tracking cancer trends, supporting research, guiding policy decisions, and ensuring efficient healthcare resource allocation. However, their effectiveness relies heavily on data quality, specifically the completeness of the cancer registration process. Background/Objectives: This systematic review aims to identify the methods used by adult population-based cancer registries worldwide to assess the completeness of their data. It also considered the significant role of high-quality data in method selection and the significant challenges of data handling. Methods: A comprehensive electronic literature search was conducted across the Web of Science, Scopus, and PubMed for studies published from January 2004 to December 2024. The review was written according to PRISMA guidelines, and the risk of bias was assessed using the Joanna Briggs Institute tool. Results: The review identified 83 studies from 31 countries, the majority from Europe, especially Nordic and Central European countries. Most studies met high-quality standards, with only one study scoring below 75%. Common approaches include independent case ascertainment (the most used), a capture–recapture analysis, a death-certificate-only analysis, and comparisons with historical rates. While independent case ascertainment is valuable when high-quality auxiliary sources are available, its accuracy varies. Other methods, like capture-recapture or Mortality-to-Incidence ratios, may be more accurate or feasible in some cases. Conclusions: The findings suggest that methods for completeness evaluations vary widely across registries, influenced by data quality and regional practices. The results emphasize the importance of the continuous refinement of these assessment methods to improve the reliability and global comparability of cancer registry data.

## Simple Summary

Accurate population-based cancer data is essential for understanding trends, guiding healthcare policies, and ensuring effective resource allocation. However, population-based cancer registries face challenges in ensuring their data is complete and reliable. The objective of this study was to evaluate what statistical methods are used worldwide to estimate cancer registry completeness, highlighting the strengths and limitations of these methods. Eighty-three studies from 31 countries were identified, with most studies meeting high-quality standards. Overall, the choice of which method to use is highly dependent on the registries’ data quality. By identifying the most effective approaches, our results emphasize the importance of continuous refinement of these assessment methods to improve the reliability and global comparability of cancer registry data. This research helps to support better decision-making in cancer prevention, treatment, and policy development, ultimately benefiting both researchers and patients.

## 1. Introduction

Population-based cancer registries (PBCRs) play a crucial role in cancer epidemiology by serving as a foundation for tracking cancer incidence over time and across different populations or subgroups of people defined by sex, age, or diagnosis [1]. By systematically collecting and analyzing data on cancer cases, these registries provide essential insights into the patterns and causes of cancer, which can then be used to design and implement health policies, support research activities, improve patient management, and reallocate resources, such as diagnostic and treatment tools [2,3]. The ability of a cancer registry to provide support to such actions strongly relies on the quality of the data fed into it [4]. In fact, the engagement of registries in such doings, with the active participation of clinicians and researchers in routine data collection and analysis, along with improved registration procedures, significantly enhance their quality [5].

Cancer registries should be evaluated based on three quality dimensions: comparability, validity, and completeness [6]. To evaluate the accuracy of a registry in estimating the true cancer incidence within a population, assessing its completeness is the most relevant dimension. It measures the degree to which all incident cancers occurring in a population are captured in the registry’s database [7].

Methods for completeness evaluations can be either qualitative (or semi-quantitative), when comparing completeness relative to other registries or over time, or quantitative, when providing numerical estimates of case registration for all eligible cases [8]. While all current methods have drawbacks and biases, ongoing efforts have been made to refine them, improving their feasibility [9]. Knowing this, PBCR worldwide struggle to find the most suitable method to assess completeness. To our knowledge, no systematic review has evaluated the methods used by population-based cancer registries to assess completeness. This study aims to identify these methods and explore challenges faced by cancer registries in data handling, how available data determine which method to use, and unveil new methods and differences in completeness assessments across different registries.

## 2. Materials and Methods

### 2.1. Search Strategy and Data Sources

The protocol for this review was registered in the International Prospective Register of Systematic Reviews (PROSPERO) on 9 July 2024 (www.crd.york.ac.uk/PROSPERO/, accessed on 7 July 2024, registration number 563033).

This study was conducted according to Cochrane Handbook for Systematic Reviews of Interventions and adheres the Preferred Reporting Items for Systematic Reviews and Meta-Analyses (PRISMA) directives to ensure transparency and reproducibility [10,11]. To locate relevant studies, an electronic literature search in the databases Web of Science https://www.webofscience.com/ (accessed on 1 July 2024), Scopus https://www.scopus.com/ (accessed on 1 July 2024), and PubMed http://www.ncbi.nlm.nih.gov/pubmed/ (accessed on 1 July 2024) was conducted. A Boolean search was conducted using the following keywords: “cancer registry” OR “cancer registries” OR “cancer registration” OR “oncologic registry”) AND “completeness”. The search was conducted between July 2024 and December 2024 as the databases continued to notify about new articles. The search was limited to recent studies published between January 2004 and up to December 2024. In addition, due to translation restrictions, only English/Portuguese/Spanish-language studies were eligible. The search strategy applied in the systematic review is presented in Table S1. A cross-reference search of all studies that met the inclusion criteria was carried out to ensure study inclusion was as complete as possible. For this, we manually examined the reference lists of the included articles.

### 2.2. Exclusion Criteria

Duplicates were identified and removed using the Desktop Mendeley software 1.19.5 version. Papers that outlined methods to assess the completeness of cancer registries were included in this study. We excluded articles that were (1) written in languages other than English, Portuguese, or Spanish; (2) repetitive or duplicated; (3) reviews, opinions, book chapters, or conference proceedings; (4) not conducted in adult population-based cancer registries; or (5) did not assess cancer registry completeness. Childhood Cancer Registries (0–14 age) were excluded as the completeness of these is usually assessed by comparing the observed age-specific rates with an expected range of values, as described in the Volume VIII of Cancer Incidence in Five Continents, by Parkin and Plummer [12]. This way, no new methods were expected to be found.

Two researchers (MPS and TM) scrutinized, independently, the titles and abstracts of the papers as of potential relevance according to the eligibility criteria. Articles included by title and abstract were reviewed in full text by the two reviewers to determine their final inclusion. A third reviewer (MJB) was asked to resolve disagreements between the two researchers during the title and abstract screening, as well as the full-text screening. The number of articles included and excluded was registered in a PRISMA flowchart—Figure S1.

### 2.3. Data Extraction and Quality Assessment

From each included study, information about the country, name of the cancer registry, years under study, and method used for the completeness assessment was gathered in Table 1, ordered by publication year. If studies reported insufficient information, we attempted to contact study authors to obtain further information, when possible; otherwise, assumptions were made based on contextual information (e.g., registry reports or methodological descriptions). All reported completeness assessment methods were collected along with the corresponding years under study, ensuring that each method was analyzed within its respective timeframe.

The risk of bias for each study was independently appraised by two review authors (MPS and TM), using the Joanna Briggs Institute’s appraisal tool specifically designed for Case Series (2020 version) [96]. To enhance the tool’s suitability to analyze the nature of the articles under review, we added other bullet points to the existing checklist, to scrutinize more effectively eventual differences between them. The following criteria were added to the tool: (1) whether the goal of the article was clearly stated; (2) if the type of registry (population-based) was clearly specified; (3) if all the cancer data sources (e.g., hospitals, death certificates, pathology reports) were explicitly mentioned and comprehensive; (4) if the completeness evaluation method was appropriate for the available data (considering some drawbacks like poor quality mortality data); and (5) if the article acknowledges any limitations in the completeness assessment methods and the generalizability of its findings. The final checklist is presented in Table S2. Studies were scored for each checklist item: “YES” (2 points), “UNCLEAR” (1 point), and “NO” (0 points), to a total of 28 points. Results were converted in a percentage, and good quality studies were those scored above 75% (21 points). The registries with worse quotation failed to report demographic information about the population under study in the methods section, providing it later in the results and discussion, so the article could be compared with other populations. In addition, there are also some articles that used population cohorts instead of the whole population, assessed completeness for a single year, or were unable to state any limitation regarding the method used for the completeness assessment and the results obtained. No attempts were made to contact study authors for additional information. All data were extracted as reported in the original publications.

## 3. Results

The initial search yielded 1619 results, of which 817 duplicates were removed prior to screening (Figure S1). From the combined 802 records, 660 additional records were excluded during screening based on the language, article type, studies not conducted in adult-population cancer registries, and not using any method to assess completeness of registries. From the remaining 142 studies, three were not retrieved leading to 139 articles for full text examinations. Upon review, 58 more results were excluded as they met the exclusion criteria. More so, one of the articles was excluded as a repetitive publication and another for being a short-version article, further substituted by its full version through cross-referencing. Overall, two articles were added through cross-referencing [13,16], which resulted in 83 studies included in this review [13,14,15,16,17,18,19,20,21,22,23,24,25,26,27,28,29,30,31,32,33,34,35,36,37,38,39,40,41,42,43,44,45,46,47,48,49,50,51,52,53,54,55,56,57,58,59,60,61,62,63,64,65,66,67,68,69,70,71,72,73,74,75,76,77,78,79,80,81,82,83,84,85,86,87,88,89,90,91,92,93,94,95].

This review comprises articles published between 2004 and December 2024 that assessed the completeness of population-based registries between 1953 and 2018. 

Most of the evaluated PBCRs belong to European countries (n = 32) with the highest percentage belonging to Nordic countries [18,24,26,27,29,30,32,36,38,39,45,48,49,53,57,59,66,70,72,76,77,80,82,85,87] (25 evaluations) and Central Europe [17,22,25,28,31,43,68,73,89,92] (7 evaluations). The set of studies includes the evaluation performed using data from 30 European countries collected in the first ENCR-JCR data call, in 2015 [88]. Among European countries, Sweden had the highest number of completeness assessments (n = 14), which started being published, periodically, since 2007 [18,24,27,36,48,49,53,57,70,72,76,77,80,85]. Following Europe, other regions assessing completeness are North America (n = 8) [14,19,42,46,54,56,74,79], the Middle East (n = 9) [51,55,58,60,63,69,81,84,95], Southeast Asia (n = 3) [34,35,62], and Oceania [16,21,33] (n = 3). For the period under study, completeness assessment studies were conducted in three countries in Africa [44,50,86], four in India [65,83,91,93], two in Australia [16,33], two in South America [40,94], and one each in New Zealand [21], Russia [90], and China [67]. The most common method to assess completeness among registries was an independent case ascertainment, being used in nearly half of the entries (n = 39) [16,18,20,21,23,24,26,27,30,32,33,36,37,38,39,45,47,48,49,53,54,56,57,59,61,66,72,74,76,77,78,79,80,82,84,85,86,87,91]. Other methods employed included the Mortality-to-Incidence ratio (n = 21) [13,15,25,28,29,31,35,39,40,52,62,65,66,67,68,73,83,88,90,92,94], historical data (n = 18) [29,31,39,44,46,50,52,62,66,67,71,73,83,90,92,93,94,95], capture–recapture (n = 15) [14,22,34,41,44,51,52,55,58,60,62,63,69,70,75,81], the flow method (n = 9) [17,29,31,41,43,47,62,64,68], death-certificate methods other than the flow method (n = 11) [13,22,34,41,42,47,52,65,68,90,95], number of notifications/sources (n = 6) [29,39,44,62,66,83], and the percentage of morphologically verified cases (n = 8) [13,31,34,42,68,88,94,95]. Additional methods exposed in this review include the age-standardized sex and cancer-site-specific incidence-to-mortality rate (IMRR) method, used by the North American Association of Central Cancer Registries (NAACCR) [19,42], and the mortality-to-incidence ratio method used by the Robert-Kock Institute to calculate the expected incidence [28,89].

Most PBCRs used a single method to assess the completeness of their registries (n = 58), while eight registries used two methods [22,65,67,71,73,88,92,93]; ten registries used three methods [13,34,41,42,44,47,83,90,94,95], five registries used four methods [19,35,40,42,85], and two registries used five methods [29,62].

The registries analyzed a diversity of neoplasms with 27 registries focusing on malignant neoplasms [13,17,20,29,39,43,47,49,52,55,62,64,65,66,67,68,71,73,75,83,86,88,90,91,93,94,95]. Fourteen of these studies excluded non-melanoma skin cancer [13,43,47,49,52,64,67,68,71,73,88,93,94,95], and three included benign or in situ neoplasms [39,49,88]. 

The majority of registries (n = 39) [14,18,20,21,24,27,28,29,30,35,36,37,39,42,44,45,46,47,48,49,53,57,59,64,65,66,68,69,70,71,73,75,76,79,87,89,90,92,95] report their neoplasms using the International Classification of Diseases (ICD7-10) [97], predominantly the 10th version (n = 32) [18,21,27,28,29,30,35,36,37,39,42,44,45,47,49,53,57,59,64,65,66,68,70,71,73,75,76,79,87,89,90,92,95]. Others opted for the International Classification of Diseases for Oncology (ICD-O 1st to 3rd edition n = 18) [31,34,38,40,50,55,56,61,62,74,78,80,83,84,85,86,88,94], recommended for tumor coding [98]. Some used both ICD9-10 and ICD-O [25,54,91,93] or the concomitant use of ICD-O only for morphology classifications [52,67]. In addition to this, there is also a mention of the use of SNOMED codes [82].

In the quality assessment, only one study received a score below 75%. Overall, the articles met high-quality standards, aligning with the established inclusion criteria. The observed high quality of the studies included may be partly due to the exclusion of gray literature.

The table compiling the results is next presented—Table 1.

## 4. Discussion

Accurate and comprehensive registry data are essential for deriving reliable cancer incidence rates, tracking trends, and guiding public health interventions. Evaluating registry completeness in adult populations is vital for developing effective cancer-control strategies and addressing the unique challenges associated with diverse cancer types and risk factors [8,12]. 

Independent case ascertainment has been employed since the early years of cancer registry development and continues to be used universally across different countries, irrespective of their economic status or geographical region—Table 1.

Most registries assess unusual patterns in incidence rates by comparing their data with independent cancer case datasets. Independent case ascertainment is widely used in Nordic countries [39,53,66], North America [54,79], and Scotland [61].

This method either involves re-screening previously registered cases or comparing registry data against external sources of cancer cases [8]. For instance, O’Brien et al. [47] used “case-finding audits” to estimate rectal cancer cases in the Irish National Cancer Registry, while Stevens et al. [21] applied similar audits to evaluate registry completeness. Although initially used to assess the completeness of specific facilities, such as hospitals, independent case ascertainment has been since adapted for population-based cancer registries [12,47]. Registry completeness is often estimated by re-examining cancer case records over a specific timeframe and identifying under-reported cases [8,99]. Lam et al. (2020) demonstrated this approach by reviewing SEER-Medicare data for Chronic Myeloid Leukemia and Bladder Cancer, using claims-based algorithms to detect missing cases through diagnosis or treatment codes [79].

Similarly, comparisons between registry data and independent sources—such as multi-center clinical trials [23], hospital discharge registries [27,39], national screening programs [47], and other cancer registries [45,53]—provide quantitative measures of completeness. Although reliable, traditional case ascertainment methods are increasingly challenged by the growing interdependence of data.

Advances in electronic health records (EHRs), as seen in the Indiana State Cancer Registry, enable direct data linkage using probabilistic and deterministic models, effectively integrating heterogeneous healthcare records [56].

Choosing the right data source for comparisons is crucial when assessing completeness. For instance, the Swedish Colorectal Cancer Registry reported over 98% completeness using the Swedish Cancer Registry as a gold standard [72]. However, since the completeness of this gold standard is not 100%, the true number of cases may have been underestimated. Similarly, Palliative Care Registries can introduce biases, as comparisons are limited to end-of-life populations [49]. The dataset size also plays a key role, as small datasets can lead to the overestimation of completeness [32].

Death certificates (DCs) are valuable for identifying cancer cases missed during life, with metrics like DCN%, DCI%, DCO%, or the DCN/M:I ratio used to assess registry completeness [13,22,34,41,42,47,52,65,68,90]. However, errors in death certificates and discrepancies between cancer diagnoses and causes of death on DCs limit their reliability, particularly for less well-defined cancers like those of the biliary tract [48,100]. Similarly, vulvar and vaginal premalignant lesions were less completely registered than tumors at the same sites, due to limited data from DCs [38].

When a death certificate cannot be matched to a registered case, it is categorized as a death certificate only (DCO). DCOs, often resulting from errors in DCs, are typically excluded as incident cases, contributing to underreporting [47]. Neither the DCO% nor DCN% accurately estimate completeness. For example, Lorez et al. demonstrated underreporting for hepatic and pancreatic cancers using DCN%, which was disproved by other methods [68]. Instead, DCO% is better interpreted as a validity measure reflecting the case-finding efficiency [95]. Despite its limitations, six studies used DCO% (n = 5) or DCN% to assess completeness but relied on complementary methods for accuracy [13,35,42,65,68]. Sharma et al. used the DCO% to evaluate the Guwahati Cancer Registry’s validity but depended on the mortality-to-incidence (M:I) ratio for a completeness assessment [65].

Another DC method calculates the proportion of cases first reported through other means than DCs. This requires identifying DCI cases to estimate unregistered but living cancer cases using M:I ratios [8]. Parkin et al. [101] introduced a formula for this purpose, later adapted by Ajiki et al. for registries with higher DCI proportions [102]. Of the five studies applying this method, four used Ajiki’s formula [41,47,52,90], while one used Parkin’s [22,41]. Schmidtmann et al. used the Lincoln–Peterson estimator, approximating Ajiki’s formula [22], while Castro et al. found comparable results using both strategies [41]. Barchuk et al. further adapted Ajiki’s index to a DCI/M:I format [90]. 

This method conceptually overlaps with capture–recapture strategies, which also use multiple data sources to assess completeness. Most studies using capture–recapture methods employed a three-source log-linear model to address interdependency and heterogeneity issues [20,34,47,55,57,58,60,61,88]. Some used Crocetti et al.’s method to evaluate dependencies [103], grouping the two most dependent sources and applying a two-source capture–recapture model with a third source [47,56,65,68,85,87]. However, this can underestimate completeness [14], as shown by Törner et al. for the Swedish Cancer Registry [70]. Schmidtmann et al. compared various capture–recapture estimators, including DCN methods, finding that all except a four-source log-linear model with second-order interactions underestimated completeness, albeit with a larger mean square error [22]. Among all methods, Parkin’s formula yielded the most accurate estimates when all cancer deaths were included. 

The aforementioned methods cannot detect unreported cases outside routine notifications, besides lacking statistical power for the early detection of incompleteness [104]. In contrast, the flow method addresses these gaps. It considers cancer registration as a post-diagnosis time-sensitive event, following a probabilistic framework to improve the completeness assessment [104]. 

For all cancer registries, completeness increased logarithmically during the first three years and stabilizes thereafter, reflecting the natural data registration process, where most cases are captured shortly after diagnosis [6,17,29,31,41,43,47,62,64,68]. However, registration delays can impact completeness assessments, particularly for tumors diagnosed in outpatient settings [68]. Nonparametric models have been proposed to address this challenge [105]. The flow method, while effective in delayed registration scenarios, is susceptible to the overestimation bias caused by registration delays [17]. To mitigate this, Bullard et al. modified the flow method adjusting it for delayed registrations [104], a strategy later applied by Fung et al. in the Singapore Cancer Registry [62]. Compared to capture–recapture methods, the flow method generally provides higher completeness estimates.

Despite its advantages, the flow method has notable limitations. It underestimates survival times, leading to an overestimation of completeness, and requires the long-term follow-up of vital status [29,43]. Additionally, it assumes stable incidence and mortality rates, and the lack of cause-of-death information on death certificates can introduce bias [47,68]. Hackl et al. criticized the method, arguing that it inadequately accounts for delayed registrations, effectively setting these cases to zero without modeling their effects [43].

Comparing death-certificate methods, O’Brien et al. found that Ajiki’s formula provides lower completeness estimates than the flow method [47], while Silcocks et al. demonstrated that the flow method is more accurate but biased for cancers with good survival rates [106]. Similarly, Schmidtmann et al. advised against using the Lincoln–Petersen estimator, an approximation of Ajiki’s formula, due to its limitations [22].

DCN methods utilizing the M:I ratio, offer a straightforward metric for a semi-quantitative completeness estimation. Of the 22 articles employing the M:I ratio, eleven exclusively relied on it [13,15,25,28,65,67,73,83,88,89,94]. For instance, Hübner et al. applied the Robert-Koch Institute methodology, comparing observed cases to expected cases based on M:I ratios from high-quality reference regions [89]. However, this method assumes consistent M:I ratios, which may not hold due to variations in screening practices, tumor stage distributions, or subtype prevalences, leading to potential inaccuracies [89,107].

Over time, the M:I ratio evolved into complementary metrics, such as 1-M:I, proposed in 1970 as both an indicator of completeness and a proxy for 5-year survival probability [107]. While useful for registries lacking survival data, its statistical validity for registry comparisons remains debated [108,109]. When mortality data are reliable and incidence and survival are stable, 1-M:I can approximate completeness [8,35]. Registries often plot the M:I ratio as a function of the 1–5-year survival rates to evaluate coherence between the variables, where deviations may suggest underreporting [29,31,35,39,40,52,62,66,68,71,90,92]. However, these interpretations depend on tumor reporting on death certificates, as well as stable incidence and mortality trends [8]. While 1-M:I has been shown to approximate 5-year survival for many tumor types, its validity as a survival proxy is questioned [109,110].

The incidence-to-mortality rate ratio (IMRR), used by NAACCR, defines completeness as the ratio of observed-to-expected incidence rates. Expected cases are derived by multiplying a registry’s mortality rate by the national IMRR, stratified by sex, race, and the cancer site. This assumes a relatively constant incidence-to-mortality ratio (I:M) across geographic areas [9]. However, Das et al. identified limitations in this assumption, proposing an enhanced model incorporating additional covariates [19], later confirmed in a Canadian study [42]. Notably, NAACCR’s indicator often produces results identical to the I:M ratio, suggesting that the latter as a simpler alternative [42].

Historical data methods are frequently used to assess registry completeness. These methods compare incidence rates among similar populations, attributing differences to local risk factors or screening practices [29,31,39,44,46,50,52,62,66,67,71,73,83,90,92,93,94,95]. Inconsistent patterns across cancer types may indicate case ascertainment issues, which can also be evaluated using age-specific incidence curves [44,52,62,71,90]. Since the incidence typically increases logarithmically with age, deviations or “drop-offs” may signal under-ascertainment [111,112]. Such patterns may also reflect socio-economic disparities or population risk factors [71].

Despite their utility in identifying trends and anomalies, historical data methods cannot provide quantitative completeness estimates, limiting their attractiveness. They are, however, useful in settings with poor-quality mortality data.

Bashar et al. compared historic methods and the M:I ratio for four Indian PBCRs in 2013, finding that completeness was underestimated by the M:I ratio at 0.27 and 0.43 times, for females and males, respectively [83]. The authors acknowledge the lack of reliable mortality data, time, and resources as reasons for using only qualitative methods, similar to Shivashankar et al. [93]. In the Icelandic Cancer Registry and Cancer Registry of Norway, incidence rates and age-specific curves were used to estimate solely the previous year of registration, also due to the unreliable mortality data [29,39].

Another metric to assess registry completeness is the percentage of cases morphologically verified (MV%), which should be compared to regional or national standards [6]. A high MV% indicates reliance on pathology laboratories and the potential inability to capture cases through alternative methods [8]. This was observed in the Basel Cancer Registry where an MV% for hepatic cancer of 94.4% was detected, against 56.7% for a Swiss PBCR [68]. This strategy strongly relies on local regional circumstances where registries are situated, being discouraged in places with inadequate or insufficient tissue processing [113]. Similar to the DCO%, this method can assess the validity and completeness at once, as demonstrated in a study on 130 European PBCRs [88].

Finally, the number of sources/notifications per case were found for six articles, as it primarily indicates how good data flow is rather than completeness [29,39,44,62,66,83]. It is interesting to notice how notification numbers vary across registries, as seen for multiple myeloma, which was low in the Norwegian registry but high in the Icelandic registry [29,39]. These variations highlight the influence of local data quality and methodological preferences on the choice of evaluation strategies.

Overall, a completeness assessment is particularly challenging for specific cancer sites, especially those with poor reporting of low morphologically verified cancers, such as those of the central nervous system, hematological malignancies, soft tissue, and pancreas [29,62,66,73,114]. Furthermore, this is more likely to occur among the elderly, for tumors with poor survival, such as lung, pancreas, or liver [27,36,49,115]. This underreporting contributes to an increasing reliance on notifications by death certificate only (DCO%) [66,73]. This highlights the importance of accurate coding on death certificates for the completeness of registries. In addition, overreporting errors may arise from the incorrect coding of sites prone to metastasis, such as the brain or liver, as primary sites [35,40], although less common in developed countries, due to improvements in diagnostic tools. Additionally, registration errors based on misclassifying close anatomical sites may also occur, as described for ureteral and bladder cancers [24].

Improved data linkages through both probabilistic and deterministic models can help fill gaps in data integration, while methods like machine learning and claims-based algorithms have the potential to discover missed cases. More so, techniques that reduce bias and delays within capture–recapture and flow techniques may increase accuracy. Reliability can be further enhanced by standardizing metrics, such as the M:I ratio, across registries and by conducting periodic audits.

The quality evaluation of studies included in this analysis highlights the robustness of the methodologies employed. Only one study received a score below 75%, reflecting overall high quality across the articles reviewed. This is likely attributable to strict inclusion criteria and the exclusion of grey literature, which often lacks rigorous peer review. Most studies met established quality standards, clearly outlining registry types, data sources, and completeness evaluation methods. However, articles with lower scores often failed to report demographic information about the study population in the methods section or assessed completeness for only a single year. Some studies did not acknowledge limitations in their methodologies, such as the use of population cohorts instead of the entire population or reliance on poor-quality mortality data. Interestingly, when looking at the four highest quality scores (25–28), of the 51 articles, almost half (23) used more than one method when assessing completeness [13,14,22,29,31,39,41,42,44,52,62,65,66,67,68,71,73,88,90,91,92,93,94]. Regarding the rest, only one method was used, with 22 articles opting for case ascertainment [18,20,21,23,26,36,37,38,48,49,53,54,59,61,74,76,77,78,79,82,86,87]. In one hand, this highlights the importance of using several methods when assessing the quality of cancer registries. On the other hand, it is clear how cancer registries find reliable independent case ascertainment, by opting for it when using only one strategy.

### Study Limitations

This systematic review has several limitations that should be considered when interpreting the findings. Excluding gray literature may have introduced publication bias, overrepresenting positive findings and underestimating the challenges associated with data completeness assessments. By restricting to English, Portuguese, or Spanish language publications and major databases (Web of Science, Scopus, and PubMed), relevant studies might have been missed, particularly impacting representation from non-European regions. The heterogeneity of included studies, despite the quality assessment, posed challenges in synthesizing the findings. The focus on what methods are used, rather than how well, limits the understanding of practical implementation. The predominantly European studies limit generalizability, especially to lower-resource settings. 

Future research should address these limitations by including gray literature, exploring additional language publications, and investigating the practical implementation and effectiveness of different completeness assessment methods across diverse settings.

## 5. Conclusions

Several statistical methods exist for evaluating cancer registry data completeness, each with its strengths and weaknesses. Common approaches include independent case ascertainment, capture–recapture analysis, death certificate-only case analysis, and comparisons with historical incidence rates. Quantitative methods are generally preferred over qualitative ones for their ability to provide precise numerical estimates, though they often require greater resources and robust data systems. The choice of most adequate method depends on specific registry’s characteristics, such as data sources and data quality, as well as the type of tumor. Enhancing data completeness requires advanced strategies and modern technologies. Crucially, the development of effective and adaptable systems for comprehensive cancer monitoring must be achieved through global cooperation among registries.

## Figures and Tables

**Table 1:** Statistical methods for the completeness of adult PBCR quality assessments, from 2004 to 2024.

Ref.	Author, Year	Country	PBCR	Years of Study	Method
[13]	Crocetti et al., 2004	Italy	Italian Network of Cancer Registries	1986–1997	MV% DCO%M:I Ratio
[14]	McClish et al., 2004	USA	Virginia Cancer Registry	1995	2—source capture–recapture 3—source capture–recapture with log-linear model
[15]	Mori et al., 2005	Japan	11 PBCRs	1994 and 1997	M:I ratio
[16]	Burgess et al., 2006	Australia	Tasmanian Cancer Registry	1988–1998	Independent case ascertainment
[17]	Montanaro et al., 2006	Switzerland	Ticino Cancer Registry	1996–2000	Flow method
[18]	Turesson et al., 2007	Sweden	Swedish Cancer registry	1964–2003	Independent case ascertainment
[19]	Das et al., 2008	USA	29 registries certified by the North American Association of Central Cancer Registries	2000	IMRR
[20]	Contiero et al., 2008	Italy	Lombardy Cancer Registry	1997–1998	Independent case ascertainment
[21]	Stevens et al., 2008	New Zealand	New Zealand Cancer Registry	2004	Independent case ascertainment
[22]	Schmidtmann, 2008	Germany	Artificial cancer registry that used information from the Rhineland-Palatinate cancer registry to estimate the real data distribution	2009	Death-certificate (DCN/M:I) methods – Lincoln-Petersen Estimator – Parkin’s formula – Capture–recapture methods – 4—Source with log–linear – Counting distribution approach – Chao and Zelterman estimators – Sample coverage estimators (M_h_, M_th_)
[23]	Brewster et al., 2008	Scotland	Scottish Cancer Registry	1978–2000	Independent case ascertainment
[24]	Holmäng et al., 2008	Sweden	Swedish Cancer Registry	1971–1998	Independent case ascertainment
[25]	Radespiel-Tröger et al., 2008	Germany	Bavarian cancer registry	2002	M:I ratio
[26]	Larjavaara et al., 2008	Finland	Finnish Cancer Registry	November 2000–June 2001	Independent case ascertainment
[27]	Barlow et al., 2009	Sweden	Swedish Cancer Registry	1998	Independent case ascertainment
[28]	Nennecke et al., 2009	Germany	Hamburg Cancer Registry’s	1995–2003	M:I ratio
[29]	Larsen et al., 2009	Norway	Cancer Registry of Norway	1953–2006	Stability of age-standardized incidence rate
				2000–2004	M:I ratio vs. 1–5-year survival rate
				2001–2005	Number of sources/notifications per case
				2001–2005	3—sources capture–recapture
				1999–2004	Flow method
[30]	Petri et al., 2009	Denmark	Danish Gynecological Cancer Database	2005–2006	Independent case ascertainment
[31]	Oberaigner et al., 2009	Austria	Cancer Registry of Tyrol	2002–2006	M:I ratio vs. 1–5-year survival rate
				1998–2006	Stability of age-standardized incidence rate
				1998–2002	MV%
				1999	Flow method
[32]	Bilet et al., 2009	Norway	Cancer Registry of Norway	1985 and 1999	Independent case ascertainment
[33]	Field et al., 2010	Australia	Victorian Cancer Registry	2000–2005	Independent case ascertainment
[34]	Suwanrungruang et al., 2011	Thailand	9 PBCRs	2003–2007	Virtual 3-source capture recapture with log-linear model DCO% MV%
[35]	Suwanrungruang et al., 2011	Thailand	5 PBCRs	2003–2007	M:I ratio vs. 1–5-year survival rate
[36]	Lambe et al., 2011	Sweden	Swedish Cancer Register	1987–1999	Independent case ascertainment
[37]	Moller et al., 2011	England	8 PBCRs	2001–2007	Independent case ascertainment
[38]	Enerly et al., 2012	Norway	Cancer Registry of Norway	2002–2007	Independent case ascertainment
[39]	Sigurdardottir et al., 2012	Iceland	Icelandic Cancer Registry	1955–2009	Stability of age-standardized incidence rate
				2005–2009	Number of sources/notifications per case
				2005–2009	M:I ratio vs. 1–5-year survival rate
				2000–2001	Independent case ascertainment
[40]	Cendales et al., 2012	Colombia	4 PBCRs	1998–2006	M:I ratio vs. 1–5-year survival rate
[41]	Castro et al., 2012	Portugal	RORENO	2001–2006	2—Sources capture–recapture Death-certificate (DCN/M:I) Flow method
[42]	Zakaria, 2013	Canada	Canadian Cancer Registry	2003–2007	IMRR
				2007	MV%
					DCO%
					Age-standardized I:M ratio
[43]	Hackl et al., 2013	Austria	Austrian National Cancer Registry	2005–2009	Flow method
[44]	Shimakawa et al., 2013	Gambia	Gambia National CancerRegistry	1990–2009	Age-specific incidence curves
					Stability age–sex–specific incidence rate
				1995–1999	Comparison of age-standardized incidence rates in different populations
				2000–2009	Number of notifications per case
					2—Sources capture–recapture
[45]	Londero et al., 2014	Denmark	Danish national clinical thyroid cancer database	1996–2008	Independent case ascertainment
[46]	Lai et al., 2014	USA	Kansas Cancer Registry	2010	Age-specific incidence rate
[47]	O’Brien et al., 2014	Republic of Ireland	Irish National Cancer Registry	2005–2009	Death-certificate (DCN/M:I) method
					Flow method
				2000–2009	Independent case ascertainment
				2007	Cancer audit
[48]	Kilander et al., 2014	Sweden	Swedish Cancer Registry	1990–2009	Independent case ascertainment
[49]	Nilsson et al., 2014	Sweden	Swedish Cancer Registry	1958–2009	Independent case ascertainment
[50]	al-Haddad et al., 2015	Nigeria	8 Nigerian PBCRs	2008–2011	Age-standardized incidence rate
[51]	Khodadost et al., 2015	Iran	Ardabil Cancer Registry	2006 and 2008	3—Sources capture–recapture with log–linear model
[52]	Dimitrova et al., 2015	Bulgaria	Bulgarian National Cancer Registry	1993–2010	Stability of incidence rates
				2003–2007	Comparison of incidence rates in different populations
					Shape of age-specific curves
				2005–2009	M:I ratio vs. 1–5-year survival rate
				2006–2010	2—Sources capture–recapture
					Death-certificate (DCN/M:I) methods
[53]	Tomic et al., 2015	Sweden	National Prostate Cancer Registry of Sweden	1998–2012	Independent case ascertainment
[54]	Tran et al., 2016	Canada	Ontario Cancer Registry	1993–2009	Independent case ascertainment
[55]	Mohammadi et al., 2016	Iran	Iranian National Cancer Registry	2008–2010	2—Sources capture–recapture
[56]	Ruppert et al., 2016	USA	Indiana State Cancer Registry	2005–2012	Independent case Aacertainment
[57]	Linder et al., 2016	Sweden	Swedish National Registry for Oesophageal and Gastric Cancer	2009–2013	Independent case ascertainment
[58]	Khodadost et al., 2016	Iran	Ardabil Cancer Registry	2006 and 2008	3-Sources capture–recapture with log-linear model
[59]	Hansen et al., 2016	Denmark	Danish Neuro-Oncology Registry	2009–2014	Independent case ascertainment
[60]	Mohammadi et al., 2016	Iran	National Iranian Cancer Registry	2008–2010	3—Sources capture–recapture with log-linear model
[61]	Morling et al., 2016	Scotland	Scottish Cancer Registry	2011–2012	Independent case ascertainment
[62]	Fung et al., 2016	Singapore	Singapore Cancer Registry	1968–2013	Stability of incidence rates
				2008–2012	Shape of age-specific curves
					M:I ratio vs. 1–5-year survival rate
					Number of notifications per case
					2—sources capture–recapture
					Flow method
[63]	Khodadost et al., 2016	Iran	Ardabil Cancer Registry	2006 and 2008	3—Sources capture–recapture with log-linear model
[64]	Donnelly et al., 2017	Northern Ireland	Northern Ireland Cancer Registry	2010–2012	Flow method
[65]	Sharma et al., 2017	India	Guwahati Cancer Registry	2009–2011 and 2012–2014	M:I ratio DCO%
[66]	Leinonen et al., 2017	Finland	Finnish Cancer Registry	1953–2013	Stability of age-standardized incidence rates
				2009–2013	Number of notifications per case
					M:I ratio vs. 1–5-year survival rate
					Independent case ascertainment
[67]	Liu et al., 2017	China	Guangzhou Cancer Registry	2004–2014	Stability of age-standardized incidence rates
				2010–2012	M:I ratio
[68]	Lorez et al., 2017	Switzerland	10 Swiss Cancer Registries	2006–2012	M:I ratio vs. 1–5-year survival rate
					MV%
				2006–2011	Flow method
					DCN%
[69]	Fararouei et al., 2017	Iran	Kohgiluyeh and Boyer-Ahmad Registry	2007–2009	3—sources capture–recapture with log-linear model
[70]	Törner et al., 2017	Sweden	Swedish Cancer Registry	1998–2010	2—sources and 3—sources capture–recapture with log-linear model
[71]	Ryzhov et al., 2018	Ukraine	National Cancer Registry of Ukraine	2002–2012	Stability of incidence rates
				2008–2012	Comparison of incidence rates with other countries
				2002–2012	Shape of age-specific curves
				2007–2011	M:I ratio vs. 1–5-year survival rate
[72]	Moberger et al., 2018	Sweden	Swedish Colorectal Cancer Registry	2008–2015	Independent case ascertainment
[73]	Wanner et al., 2018	Switzerland	Cancer Registry Zurich and Zug	1981–2014	Stability of incidence rates
				1980–2014	M:I ratio
[74]	Eckstrand et al., 2018	Canada	Alberta Cancer Registry	2010–2015	Independent case ascertainment
[75]	Plouvier et al., 2019	France	Lille Area Cancer Registry	2008–2009	3—Sources capture–recapture with log-linear method
[76]	Tettamanti et al., 2019	Sweden	Swedish Cancer Registry	1990–2014	Independent case ascertainment
[77]	Löfgren et al., 2019	Sweden	National Breast Cancer Register of Sweden	2010–2014	Independent case ascertainment
[78]	van der Willik et al., 2020	Netherlands	Netherlands Cancer Registry	1989–2012	Independent case ascertainment
[79]	Lam et al., 2020	USA	SEER-18 registry	2008–2015 and 2007–2015	Independent case ascertainment
[80]	Lambe et al., 2020	Sweden	Swedish Cancer Registry	2013	Independent case ascertainment
[81]	Etemad et al., 2020	Iran	Khuzestan Province Cancer Registry	2011	3—Sources capture–recapture with log-linear model
[82]	Danckert et al., 2020	Denmark	Danish Renal Cancer Group Database	1 August 2010–31 December 2015	Independent case ascertainment
[83]	Bashar et al., 2021	India	4 PBCRs	2013	Comparison of stability of incidence rates in different populations M:I ratio Number of sources per case
[84]	Moore et al., 2021	Israel	Israel National Cancer Registry	2005	Independent case ascertainment
[85]	Landberg et al., 2021	Sweden	National Swedish Kidney Cancer Register	2008–2017	Independent case ascertainment
[86]	Somdyala et al., 2021	South Africa	Eastern Cape Cancer Registry	2014–2015	Independent case ascertainment
[87]	Maharjan et al., 2022	Finland	Finnish Cancer Registry	1990–2014	Independent case ascertainment
[88]	Giusti et al., 2023	30 European Countries	130 PBCRs	1995–2014	MV% M:I ratio
[89]	Hübner et al., 2023	Germany	German Federal States Cancer Registries	1999–2015	Robert Koch Institute method
[90]	Barchuk et al., 2023	Russia	10 PBCRs	2013–2017	M:I ratio vs. 1–5-year survival rate
				1993–2017	Stability of incidence rates over time
				2008–2017	Shape of Age-specific curves
					Lincoln–Petersen estimator
					Death-certificate (DCI/M:I) methods
[91]	Swaminathan et al., 2023	India	Dindigul Ambilikkai Cancer Registry	2003–2010	Independent case Aacertainment
[92]	Wéber et al., 2023	Hungary	Hungarian National Cancer Registry	2000–2019	Stability of age-standardized incidence rates
				2014–2018	M:I ratio vs. 1–5-year survival rate
				2018	Age-standardized incidence rates (ASRs) per 100,000 person-years
				2012–2018	Stability of age-standardized incidence rates
[93]	Shivshankar et al., 2024	India	Ratnagiri Cancer Registry	2017–2018	Stability of incidence rates over time Comparison of age-adjusted incidence Rates average number of sources per case
[94]	E Silva et al., 2024	Brazil	33 PBCRs	2000–2018	Stability of age-standardized incidence rates M:I ratio MV%
[95]	Lotfi et al., 2024	Iran	Isfahan Cancer Registry	2015–2018	Stability of age-standardized incidence Rates MV% DCO%

## Data Availability

No new data were created or analyzed in this study. Data sharing is not applicable to this article.

## Supplementary Materials

The following supporting information can be downloaded at: https://www.mdpi.com/article/10.3390/cancers17071123/s1, Table S1: Search Strategy; Figure S1: Preferred Reporting Items for Systematic Reviews and Meta-Analyses (PRISMA) flowchart of study selection, with search strategy; Table S2: Articles quality assessment using the Joanna Briggs Institute’s appraisal tool for Case Series with additional inquiries.

## Abbreviations

The following abbreviations are used in this manuscript:

DCI%	Percentage of Death Certificate Initiated
DCN%	Percentage of Death Certificate Notification
I:M ratio	Incidence-to-Mortality ratio
IMRR	Incidence-to-mortality rate ratio
M:I ratio	Mortality-to-incidence ratio
MV%	Percentage of Microscopically Verified Tumors
PBCR	Population-based Cancer Registry
