# Supplementary material for: Completeness Evaluation of Adult-Population-Based Cancer Registries: A Systematic Review
Source: Cancers (Basel). 2025 Mar 27;17(7):1123. doi: 10.3390/cancers17071123 (PMC11988001; doi:10.3390/cancers17071123)
Supplement: Supplementary file 1 [file cancers-17-01123-s001.zip › cancers-3511395-supplementary.pdf]

Completeness Evaluation of Adult-Population-Based Cancer Registries: A Systematic Review

Mariana P. Sousa, Teresa Monjardino, Cristina Costa Santos, Lúcio Lara, Maria José Bento

Table S1. Search Strategy.

Database	Search Details
**Pubmed**	("cancer registry" OR "cancer registries" OR "cancer registration" OR "oncologic registry") AND "completeness" AND ("2004/01/01"[Date - Entry] : "3000"[Date - Entry])
**Scopus**	((cancer registration) AND (completeness) AND (method)) AND ((cancer) AND (registry) AND (methods) AND (completeness))
**Web of Science**	(ALL=(("cancer registry" OR "cancer registries" OR "cancer registration" OR "oncologic registry" OR "cancer database") AND "completeness") and 2024 or 2023 or 2022 or 2021 or 2020 or 2019 or 2018 or 2017 or 2016 or 2015 or 2014 or 2013 or 2012 or 2011 or 2010 or 2009 or 2008 or 2007 or 2006 or 2005 or 2004 (Publication Years))

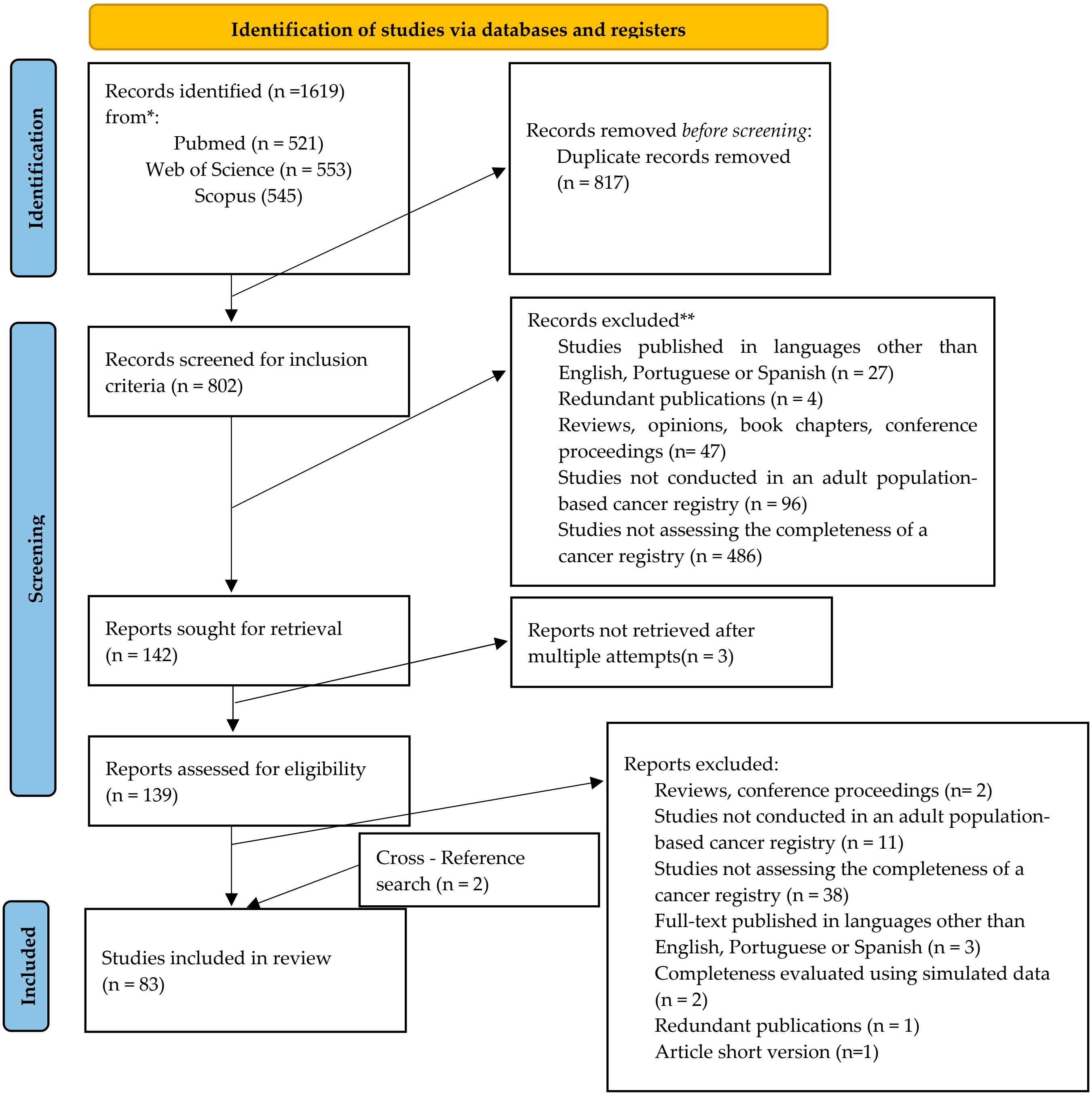

Figure S1. Preferred Reporting Items for Systematic Reviews and Meta-Analyses (PRISMA) flowchart of study selection, with search strategy.

Table S2. Articles quality assessment using the Joanna Briggs Institute's appraisal tool for Case Series with additional inquiries.

	Were there clear criteria for inclusion in the case series?	Is the objective of the article clearly stated?	Was the condition measured in a standard, reliable way for all participants included in the case series?	Were valid methods used for identification of the condition for all participants included in the case series?	Did the case series have consecutive inclusion of participants?	Did the case series have complete inclusion of participants?	Was there clear reporting of the demographics of the participants in the study?	Was there clear reporting of clinical information of the participants?	Was the type of registry (population-based) clearly specified?	Are the sources of cancer data (e.g., hospitals, death certificates) explicitly mentioned and are they comprehensive?	Were the outcomes or follow up results of cases clearly reported?	Was there clear reporting of the presenting site(s)/clinic(s) demographic information?	Was statistical analysis appropriate?	Was the method appropriate to the available data?	Does the article acknowledge any limitations of the completeness assessment methods and the generalizability of the findings?	Final Score
**Crocetti, 2004**	NA	2	2	1	2	2	2	2	1	2	2	2	2	2	2	26
**McClish *et al.*, 2004**	NA	2	2	2	0	2	2	2	2	2	2	2	2	1	2	25
**Mori *et al.*, 2005**	NA	2	2	2	0	2	2	0	2	2	2	1	2	1	2	22
**Burgess *et al.*,2006**	NA	1	2	2	2	2	0	2	2	2	2	1	2	2	0	22
**Montanaro *et al.*, 2006**	NA	2	2	2	2	2	1	1	2	2	2	0	2	2	2	24
**Turesson *et al.*, 2007**	NA	2	2	2	2	2	2	2	2	2	2	2	2	2	2	28
**Das *et al.*, 2008**	NA	2	2	2	0	2	2	1	2	2	2	2	2	2	2	25
**Contiero *et al.*,2008**	NA	2	2	2	2	2	0	2	2	2	2	1	2	2	2	25
**Stevens *et al.*, 2008**	NA	2	2	2	0	2	1	2	2	2	2	2	2	2	2	25
**Schmidtmann, 2008**	NA	2	2	2	0	2	1	2	2	2	2	2	2	2	2	25
**Brewster *et al.*, 2008**	NA	2	2	2	2	2	2	2	2	2	2	2	2	2	2	28
**Holmäng *et al.*, 2008**	NA	2	2	2	2	2	1	2	2	2	2	1	2	2	0	24
**Radespiel-Tröger *et al.*, 2008**	NA	2	2	2	0	2	2	2	2	2	2	2	1	1	2	24
**Larjavaara *et al.*, 2008**	NA	2	2	2	2	2	2	2	2	2	2	2	2	2	2	28
**Barlow *et al.*, 2009**	NA	2	2	2	0	0	2	2	2	2	2	2	2	2	2	24
**Nennecke *et al.*, 2009**	NA	2	2	2	2	2	2	2	2	1	2	1	2	1	2	25
**Larsen *et al.*, 2009**	NA	2	2	2	2	2	1	2	2	2	2	2	2	2	2	27
**Petri *et al.*, 2009**	NA	1	2	2	2	2	1	2	1	2	2	1	2	2	2	24
**Oberaigner *et al.*, 2009**	NA	2	2	2	2	2	1	2	2	2	2	2	2	2	2	27
**Bilet *et al.*, 2009**	NA	2	2	2	0	2	1	2	2	2	2	0	2	2	2	23
**Field *et al.*, 2010**	NA	1	2	2	2	2	1	2	2	2	2	0	2	2	2	24

**Suwanrungruang *et al.*, 2011**	NA	2	2	2	2	2	2	1	2	2	2	0	2	1	2	24
**Suwanrungruang *et al.*, 2011**	NA	2	2	2	2	2	1	2	2	2	2	1	2	0	2	24
**Lambe *et al.*, 2011**	NA	2	2	2	2	2	1	2	2	2	2	2	2	2	2	27
**Moller *et al.*, 2011**	NA	2	2	2	2	2	1	2	2	2	2	2	2	2	2	27
**Enerly *et al.*, 2012**	NA	2	2	2	2	2	1	2	2	2	2	1	2	2	2	26
**Sigurdardottir *et al.*, 2012**	NA	2	2	2	2	2	1	2	2	2	2	2	2	2	1	26
**Cendales *et al.*, 2012**	NA	2	2	2	2	2	0	2	2	2	2	2	2	2	2	26
**Castro *et al.*, 2012**	NA	2	2	1	2	2	1	2	2	2	2	1	2	2	2	25
**Zakaria, 2013**	NA	2	2	2	2	2	2	2	2	2	2	2	2	2	2	28
**Hackl *et al.*, 2013**	NA	2	2	2	2	2	0	2	2	2	2	0	2	1	2	23
**Shimakawa *et al.*, 2013**	NA	2	2	2	2	2	1	2	2	2	2	2	2	2	2	27
**Londero *et al.*, 2014**	NA	2	2	2	2	2	1	2	2	2	2	1	2	2	0	24
**Lai *et al.*, 2014**	NA	2	2	2	0	2	0	2	2	2	2	2	2	2	2	24
**O'Brien *et al.*, 2014**	NA	2	2	1	2	2	2	2	2	2	1	2	1	1	2	24
**Kilander *et al.*, 2014**	NA	2	2	2	2	2	2	2	2	2	2	2	2	2	2	28
**Nilsson *et al.*, 2014**	NA	2	2	2	2	0	1	2	2	2	2	2	2	2	2	25
**al-Haddad *et al.*, 2015**	NA	2	2	2	2	2	0	1	2	2	2	1	2	2	2	24
**Khodadost *et al.*, 2015**	NA	2	2	2	0	2	0	2	2	2	2	2	2	1	1	22
**Dimitrova *et al.*, 2015**	NA	2	2	2	2	2	0	2	2	2	2	2	2	2	2	26
**Tomic *et al.*, 2015**	NA	2	2	2	2	2	1	2	2	2	2	1	2	2	2	26
**Tran *et al.*, 2016**	NA	2	2	2	2	2	0	2	2	2	2	1	2	2	2	25
**Mohammadi *et al.*, 2016**	NA	2	2	2	2	2	0	2	2	2	2	1	2	2	0	23
**Ruppert *et al.*, 2016**	NA	2	2	2	2	2	1	2	2	2	2	1	2	2	0	24
**Linder *et al.*, 2016**	NA	2	2	2	2	0	1	2	2	2	2	1	2	2	1	23
**Khodadost *et al.*, 2016**	NA	2	2	2	2	0	0	2	2	2	2	2	2	2	1	23
**Hansen *et al.*, 2016**	NA	2	2	2	2	2	2	2	2	2	2	2	2	2	2	28
**Mohammadi *et al.*, 2016**	NA	2	2	2	2	2	0	2	2	2	2	2	2	2	0	24
**Morling *et al.*, 2016**	NA	2	2	2	2	2	2	2	2	2	2	2	2	2	2	28
**Fung *et al.*, 2016**	NA	2	2	2	2	2	0	2	2	2	2	2	2	2	2	26
**Khodadost *et al.*, 2016**	NA	2	2	2	2	0	0	2	2	2	2	2	2	2	1	23
**Donnelly *et al.*, 2017**	NA	2	2	2	2	2	0	2	2	2	2	2	2	2	1	25
**Sharma *et al.*, 2017**	NA	2	2	2	2	2	2	2	2	2	2	2	2	0	2	26
**Leinonen *et al.*, 2017**	NA	2	2	2	2	2	0	2	2	2	2	2	2	2	1	25
**Liu *et al.*, 2017**	NA	2	2	2	2	2	0	2	2	2	2	2	2	2	1	25
**Lorez *et al.*, 2017**	NA	2	2	2	2	2	0	2	2	2	2	1	2	2	2	25
**Fararouei *et al.*, 2017**	NA	2	2	2	2	2	0	2	2	2	2	1	2	2	2	25
**Törner *et al.*, 2017**	NA	2	2	2	2	2	0	2	2	2	1	1	2	2	2	24
**Ryzhov *et al.*, 2018**	NA	2	2	2	2	2	2	2	2	2	2	2	2	2	2	28

**Moberger *et al.*, 2018**	NA	2	2	2	2	0	0	2	2	2	2	0	2	2	1	21
**Wanner *et al.*, 2018**	NA	2	2	2	2	2	1	2	2	2	2	2	2	2	2	27
**Eckstrand *et al.*, 2018**	NA	2	2	2	2	2	2	2	2	2	2	2	2	2	2	28
**Plouvier *et al.*, 2019**	NA	2	2	2	2	2	2	2	2	2	2	2	2	2	1	27
**Tettamanti *et al.*, 2019**	NA	2	2	2	2	2	1	2	2	2	2	2	2	2	2	27
**Löfgren *et al.*, 2019**	NA	2	2	2	2	2	1	2	2	2	2	2	2	2	0	25
**van der Willik *et al.*, 2020**	NA	2	2	2	2	2	1	2	2	2	2	2	2	2	2	27
**Lam *et al.*,2020**	NA	2	2	2	2	2	1	2	2	2	2	1	2	2	2	26
**Lambe *et al.*, 2020**	NA	2	2	2	0	2	0	2	2	2	1	1	2	2	2	22
**Etemad *et al.*, 2020**	NA	2	2	2	0	2	0	2	2	2	2	2	2	2	2	24
**Danckert *et al.*, 2020**	NA	2	2	2	2	2	1	2	2	2	2	2	2	2	1	26
**Bashar *et al.*, 2021**	NA	2	2	2	0	2	0	2	2	2	2	1	2	2	1	22
**Moore *et al.*, 2021**	NA	2	2	2	0	2	0	2	2	2	2	1	2	2	2	23
**Landberg *et al.*, 2021**	NA	2	2	2	2	2	0	2	2	2	2	0	2	2	0	22
**Somdyala *et al.*, 2021**	NA	2	2	2	2	2	0	2	2	2	2	1	2	2	2	25
**Maharjan *et al.*, 2022**	NA	2	2	2	2	2	1	2	2	2	2	2	2	2	1	26
**Giusti *et al.*, 2023**	NA	2	2	2	2	2	2	2	2	2	2	2	2	2	2	28
**Hübner *et al.*, 2023**	NA	2	2	2	2	2	1	2	2	2	1	1	2	2	1	24
**Barchuk *et al.*, 2023**	NA	2	2	2	2	2	2	2	2	2	2	2	2	2	2	28
**Swaminathan *et al.*, 2023**	NA	2	2	2	2	2	1	2	2	2	2	2	2	2	1	26
**Wéber *et al.*, 2023**	NA	2	2	2	2	2	1	2	2	2	2	2	2	2	0	25
**Shivshankar *et al.*, 2024**	NA	2	2	2	2	2	0	2	2	2	2	2	2	2	2	26
**E Silva *et al.*, 2024**	NA	2	2	2	2	2	1	2	2	2	2	2	2	2	2	27
**Lotfi *et al.*, 2024**	NA	2	2	1	2	2	2	2	2	2	0	2	2	2	0	23
